# Alkyl hydroperoxide reductase enhances the growth of *Leuconostoc mesenteroides* lactic acid bacteria at low temperatures

**DOI:** 10.1186/s13568-015-0098-3

**Published:** 2015-02-18

**Authors:** Seitaro Goto, Jun Kawamoto, Satoshi B Sato, Takashi Iki, Itaru Watanabe, Kazuyuki Kudo, Nobuyoshi Esaki, Tatsuo Kurihara

**Affiliations:** Product Development Laboratory, NH Foods Ltd., Chikusei, Ibaraki 308-0042 Japan; Institute for Chemical Research, Kyoto University, Uji, Kyoto 611-0011 Japan; Research Center for Low Temperature and Materials Sciences, Kyoto University, Kyoto, 606-8501 Japan

**Keywords:** Lactic acid bacteria, Food spoilage, Cold adaptation, AhpC

## Abstract

Lactic acid bacteria (LAB) can cause deterioration of food quality even at low temperatures. In this study, we investigated the cold-adaptation mechanism of a novel food spoilage LAB, *Leuconostoc mesenteroides* NH04 (NH04). *L. mesenteroides* was isolated from several spoiled cooked meat products at a high frequency in our factories. NH04 grew rapidly at low temperatures within the shelf-life period and resulted in heavy financial losses. NH04 grew more rapidly than related strains such as *Leuconostoc mesenteroides* NBRC3832 (NBRC3832) at 10°C. Proteome analysis of NH04 demonstrated that this strain produces a homolog of alkyl hydroperoxide reductase––AhpC––the expression of which can be induced at low temperatures. The expression level of AhpC in NH04 was approximately 6-fold higher than that in NBRC3832, which was grown under the same conditions. Although AhpC is known to have an anti-oxidative role in various bacteria by catalyzing the reduction of alkyl hydroperoxide and hydrogen peroxide, the involvement of AhpC in cold adaptation of food spoilage bacteria was unclear. We introduced an expression plasmid containing *ahpC* into NBRC3832, which grows slower than NH04 at 10°C, and found that expression of AhpC enhanced growth. These results demonstrated that AhpC, which likely increases anti-oxidative capacity of LAB, plays an important role in their rapid growth at low temperatures.

## Introduction

Lactic acid bacteria (LAB) are useful for the production of fermented foods such as soy sauce and cheese (Caplice and Fitzgerald 1999; McKay and Baldwin 1990; Murooka and Yamshita 2008). Although the importance of LAB is widely recognized, LAB sometimes cause quality defects in fresh foods and fermented meat products stored at low temperatures (Borch et al. 1996). LAB that cause food spoilage can grow and cause quality defects by producing an unpleasant taste, white liquid, and a slimy substance (Asano et al. 2009; Hamasaki et al. 2003; Kondo and Ikeda 2000) even in cooked meat products such as ham and sausage stored at 10°C. Therefore, to ensure the safety of stored food and reduce such risks, elucidation of the cold-adaptation mechanism of LAB in food products is important. LAB that are closely related to *Leuconostoc mesenteroides*, *Lactococcus lactis*, *Leuconostoc citreum,* and *Weissella viridescens* grow rapidly at temperatures below 10°C (Borch et al. 1996; Chenoll et al. 2007; Diez et al. 2009; Hamasaki et al. 2003; Metaxopoulos et al. 2002; Samelis et al. 2006; Samelis et al. 1998), and are frequently isolated from spoiled cooked meat products stored at 10°C. These findings suggest a unique system of adaptation to low temperatures, causing rapid food spoilage in the refrigerator.

At low temperatures, microbes must overcome unfavorable conditions such as decreased membrane fluidity, molecular dynamics, and enzymatic activities. They can adapt to such an extreme environment via the induction of cold-shock and cold-acclimation proteins responsible for the maintenance of membrane fluidity, protein synthesis and folding, and metabolism (Feller and Gerday 2003; Graumann and Marahiel 1998; Marceau et al. 2004; Salotra et al. 1995; Wang et al. 2005; Wouters et al. 2000; Yamanaka et al. 1998). Identification and characterization of these proteins is necessary to understand the environmental adaptations of psychrotrophic LAB. Many studies on cold-inducible proteins have been reported, but few have focused on the relationship between these proteins and the growth of food spoilage bacteria. In order to prevent the contamination of foods by psychrotrophic LAB and to develop a method for their detection, we focused on the cold-adaptation mechanism of LAB at the molecular level. In this study, we characterized a novel psychrotrophic LAB, *Leuconostoc mesenteroides* NH04 (NH04), isolated from cooked meat stored at 10°C. *L. mesenteroides* was isolated from several spoiled cooked meat products at high frequency in our factories. NH04 grows rapidly at low temperatures within the shelf-life period and is responsible for the significant financial losses incurred. We analyzed the proteins that were inducibly expressed by this strain at low temperatures and identified a protein that facilitates the growth of *L. mesenteroides* at low temperatures.

## Materials and methods

### Bacterial strains, plasmids, and culture conditions

The strains used in this study are NH04 isolated from spoiled sausage and the related LAB, *Leuconostoc mesenteroides* NBRC3832 (NBRC3832) (NITE Biological Resource Center; Kisarazu, Japan). NH04 was deposited in NITE Biological Resource Center with accession number NBRC110676. For isolation of NH04, 25 g of spoiled product was suspended in 225 mL saline, and the suspension was subjected to 10-fold serial dilution. The dilutions (1 mL) were mixed with 15–20 mL BCP medium containing agar (Nissui Pharmaceutical Co., Ltd.; Tokyo, Japan), and the plates were incubated at 25°C for 72 h. The dominant species in the plates were isolated, and identified by sequencing their 16S rRNA genes. The nucleotide sequence of the 16S rRNA gene of NH04 was deposited in DDBJ with accession number LC005518. NH04 thus isolated and NBRC3832 were grown in 5 mL GAM broth (Nissui Pharmaceutical Co., Ltd.), with 1.0% d-glucose added to improve LAB growth. GAM broth consisted of peptone (10.0 g), soy peptone (3.0 g), proteose peptone (10.0 g), digested serum (13.5 g), yeast extract (5.0 g), meat extract (2.2 g), liver extract (1.3 g), dextrose (3.0 g), potassium dihydrogen phosphate (2.5 g), sodium chloride (3.0 g), soluble starch (5.0 g), l-cysteine hydrochloride (2.5 g), and sodium thioglycolate (0.3 g) dissolved in 1 L of deionized water (final pH of 7.1). The cells were grown at 10°C or 25°C in a compact rocking incubator (TVS062CA; ADVANTEC Toyo; Tokyo, Japan) by shaking at 70 rpm, and the growth was simultaneously monitored by measuring the turbidity at 660 nm with a spectrophotometer installed in the incubator. The kinetics of the growth was determined from three independent experiments. The LAB shuttle vector pGK::*nuc*MCS (Le Loir et al. 1994) was used for the transformation of NBRC3832. Transformants harboring pGK::*nuc*MCS and pGK*ahp*C, which was constructed as described below, were cultivated in the presence of erythromycin (5 μg/mL).

### Identification of cold-inducible proteins by two-dimensional electrophoresis (2DE)

Cells (10 mL) grown to stationary phase (1.5 < OD_600_ < 2.5) at 10°C and 25°C were harvested by centrifugation and resuspended in 100 μl of 50 mM Tris–HCl (pH 7.0). Cell suspensions were sonicated for 50 min at 10% amplitude (3 mm microtip, 400 W; Digital Sonifier; BRANSON Ultrasonics Corporation; Danbury, CT) while they were chilled on ice, and then centrifuged at 2,200 × *g* for 10 min at 4°C. Proteins in the supernatants were purified using the ReadyPrep 2-D Cleanup Kit (Bio-Rad Laboratories, Inc.; Hercules, CA) before an initial isoelectric focusing. Proteins (150 μg) were loaded onto Ready Strip IPG strips (17 cm, pH 4–7; Bio-Rad Laboratories, Inc.), and isoelectric focusing was performed using the PROTEAN IEF Cell (Bio-Rad Laboratories, Inc.), according to the manufacturer’s protocol. Treatment of the gel strips for 2DE was carried out as described previously (Kawamoto et al. 2007). After fixation and staining with SYPRO Ruby (Invitrogen Corp.; Carlsbad, CA), gels were scanned using Typhoon 9400 image analyzer (GE Healthcare Ltd.; Buckinghamshire, UK). Each experiment was performed three times to ensure reproducibility.

N-Terminal amino acid sequence of the protein inducibly produced at 10°C was determined using a PPSQ-21 protein sequencer (Shimadzu; Kyoto, Japan). A BLAST search (http://blast.ncbi.nlm.nih.gov/Blast.cgi) with the identified N-terminal sequence suggested that the cold-inducible protein was a homolog of AhpC. Degenerate primers for amplification of the *ahpC* gene of NH04 [peroxi130_For and peroxi569_Rev (Table 1)] were designed from conserved regions of the genes coding for AhpC from several LAB, including *Leuconostoc, Lactobacillus*, and *Enterococcus* species, and the internal nucleotide fragment was amplified. Based on the internal sequence of the gene coding for AhpC in NH04, an inverse PCR was performed with two primers (peroxi232_inv_For and peroxi407_inv_Rev) listed in Table 1. The sequences of the PCR products were determined and assembled to obtain the complete sequence of *ahpC*, which was deposited in DDBJ with accession number AB819067.

**Table 1:** **Primers used in this study**

**Primer**	**Sequence**
peroxi130_For	GACTTCTCATTTGTTTGYCC
peroxi569_Rev	TAMAKYTTRCCRACTARRTCWARRCT
peroxi232_inv_For	GTATCCGTAGCTTCTGCC
peroxi407_inv_Rev	CATATACCATCAACAACATGGG
peroxi_start_For	ATGACTACAAATTTTATTGATTCAGAAATAACAGA
peroxi_end_SalI_Rev	ACGCGTCGACGTCCTAAATTTTACCAACTAAATCTAAGCTCG
thr_prm_XhoI_For	CCGCTCGAGCGGATCATCTGATAGATATCGATCATAAGAG
thr_prm_peroxi_Rev	TCAATAAAATTTGTAGTCATGATTAATTCTCCTTTTTTGTGACAAAAGTA

### RNA extraction and quantitative real-time RT-PCR

Total RNA was extracted from cells cultivated at 10°C and 25°C using the RNeasy Kit (QIAGEN Inc., Valencia, CA). RNA pellets were dissolved in 0.1% diethyl pyrocarbonate-treated water and stored at −80°C until use. Quantitative real-time RT-PCR was performed with SuperScript III Platinum SYBR Green One-Step qRT-PCR Kit (Invitrogen Corp.) and an Mx3000P Multiple Quantitative RT-PCR system (Stratagene; La Jolla, CA). The amount of mRNA in each sample was normalized with the amount of 16S rRNA. Each experiment was performed three times to ensure reproducibility.

### Construction of AhpC-overexpressing strain

A gene fragment coding for AhpC was amplified using the primers peroxi_start_For and peroxi_end_SalI_Rev (Table 1), and the PCR product was fused with a DNA fragment containing a putative promoter region of the NH04 mannitol dehydrogenase gene (*mdh*) amplified by PCR with the primers thr_prm_XhoI_For and thr_prm_peroxi_Rev. The gene fragment was ligated with the pGK::*nuc*MCS plasmid after digestion with XhoI and SalI. The constructed plasmid, pGK*ahp*C, was introduced into NBRC3832 and NH04 by electroporation. Electroporation was performed using a previously described method with slight modifications (Leathers et al. 2004). Briefly, the cells from 50 mL culture were harvested at an OD_600_ of 0.6, washed twice with 75 mL of ice-cold deionized water, then washed once with 5 mL of ice-cold sterile electroporation buffer (1 mM of potassium phosphate buffer (pH 7.4) containing 1 mM of MgCl_2_ and 0.5 M of sucrose), and suspended in 1 mL of electroporation buffer. Plasmids (1 μg) were mixed with 40 μL of the cell suspensions and placed into a pre-chilled electroporation cuvette (0.2 cm). Electroporation was performed with the Gene Pulser Xcell PC system (Bio-Rad Laboratories, Inc.) at a capacitance of 25 μF. After electroporation, the cell suspensions were immediately diluted with 1 mL of GAM broth containing 1% d-glucose and incubated at 25°C for 2 h. Transformants were selected on GAM plates containing 1% d-glucose and 5 μg/mL of erythromycin.

Production of AhpC from NH04 was analyzed by SDS-PAGE. SDS-PAGE was performed on a 12.5% polyacrylamide gel (ePAGEL; ATTO Corp.; Tokyo, Japan). Soluble proteins (5 μg) were loaded and run on the gel. After fixation and staining with SYPRO Ruby, the gels were scanned using a Typhoon 9400 imager.

## Results

### Growth of NH04

**Figure 1:**
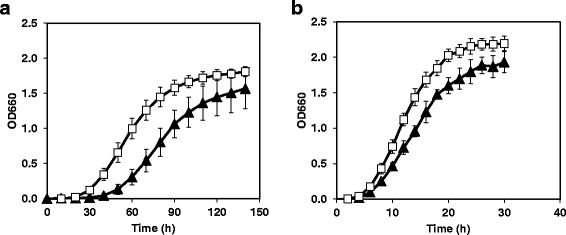
**Growth of NH04 and NBRC3832.** NH04 (square) and NBRC3832 (triangle) were cultured at 10°C (a) and 25°C (b). Each growth curve was plotted using data obtained from three experiments.

The growth of NH04 and NBRC3832 at 10°C and 25°C was monitored. NH04 grew faster than NBRC3832 at both 10°C and 25°C (Figure 1a, b). The doubling times of NH04 were 9 ± 0.6 h and 1.8 ± 0.1 h at 10°C and 25°C, respectively, whereas those of NBRC3832 were 13 ± 0.7 h and 2.5 ± 0.3 h at 10°C and 25°C, respectively. These results indicate that there were significant differences (*p* < 0.05, n = 3) between the doubling times of the two strains at both 10°C and 25°C.

### Identification of the cold-inducible protein of cold-adapted *Leuconostoc mesenteroides* NH04

**Figure 2:**
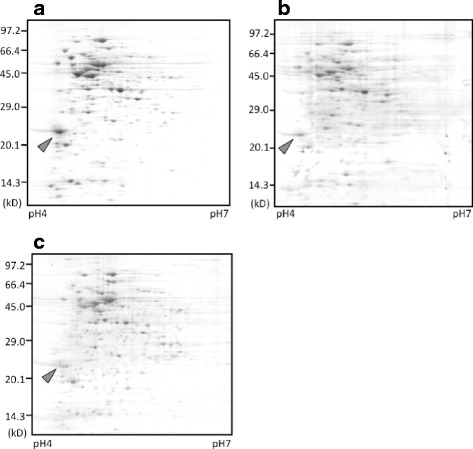
**Two-dimensional polyacrylamide gel electrophoresis of soluble proteins extracted from NH04 grown at 10°C** **(a) and 25°C** **(b) and the related strain, NBRC3832, grown at 10°C** **(c).** Arrowhead indicates the spot corresponding to AhpC.

To identify the proteins contributing to the cold adaptation of NH04, we performed 2DE of the soluble proteins of NH04 grown at 10°C and 25°C. 2DE analysis showed that the amount of a specific protein, indicated by arrowhead in Figure 2a and b, was 2 ± 0.2 times higher (*p* < 0.05, n = 3) in the cells grown at 10°C than in the cells grown at 25°C. We further characterized this protein because it was one of the major proteins of NH04 and reproducibly more abundant at 10°C. The N-terminal amino acid sequence of this protein was MTTNFIDSEITDFKVNAYHD. The gene encoding this protein was cloned and sequenced as described in Materials and Methods, and it was found that this cold-inducible protein was a homolog of alkyl hydroperoxide reductase (AhpC). The *ahpC* gene (AB819067) coded for a protein of 189 amino acid residues, and the calculated molecular weight of the protein was 21,118.5. AhpC of NH04 shares a high sequence similarity (99.5% identity) with that of NBRC3832 (AB921977). Despite the high sequence similarity, the expression level of this protein was much higher in NH04 than in NBRC3832. 2DE analysis of the soluble proteins of NBRC3832 grown under the same conditions demonstrated that the fluorescence intensity of the spot for AhpC (arrowhead in Figure 2c) was approximately 17% ± 12% of that for NH04 (arrowhead in Figure 2a).

### Transcriptional levels of *ahpC* in *Leuconostoc* species

The transcriptional level of *ahpC* in the NH04 cells grown at 10°C was approximately 3.8 ± 1.5-fold greater (*p* < 0.05, n = 3) than that in the cells grown at 25°C. The level of *ahpC* in NH04 grown at 10°C was approximately 2.8 ± 0.4-fold higher (*p* < 0.05, n = 3) than that in NBRC3832 grown under the same conditions.

### AhpC facilitates the growth of LAB at low temperatures

**Figure 3:**
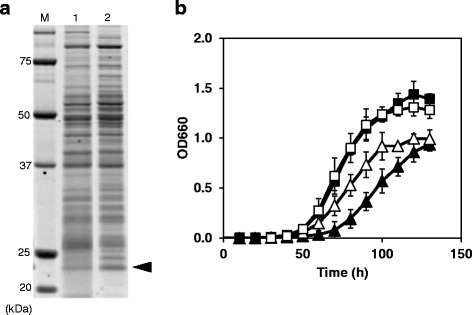
**Effects of** ***ahpC*** **overexpression on the growth of NBRC3832. (a)** Production of AhpC from NH04 in NBRC3832. Soluble proteins from NBRC3832 harboring a control plasmid (lane 1) and an *ahpC* overexpression plasmid (lane 2) were analyzed by SDS-PAGE. Arrowhead indicates the band corresponding to AhpC. Lane M represents the molecular weight marker. **(b)** Growth of NH04 (square) and NBRC3832 (triangle) containing a control plasmid (filled symbol) and an *ahpC* overexpression plasmid (open symbol) at 10°C. Each growth curve was plotted using data obtained from three experiments.

To examine the effects of high-level expression of AhpC on the growth of LAB at low temperatures, we constructed an overexpression vector for *ahpC* (pGK*ahp*C) and introduced it into NBRC3832, which grows slower than NH04. The level of AhpC in NBRC3832 harboring pGK*ahp*C was approximately 6.5 ± 1.6-fold higher than that in the cells harboring a control plasmid, pGK::*nuc*MCS (Figure 3a). NBRC3832 overexpressing *ahpC* grew more rapidly than the cells containing a control plasmid at 10°C (Figure 3b). The doubling time of NBRC3832 harboring pGK*ahp*C was 15.4 ± 0.8 h, whereas that of the strain harboring pGK::*nuc*MCS was 17.6 ± 1.1 h. The difference between the growth rates of NBRC3832 harboring pGK*ahp*C and pGK::*nuc*MCS was statistically significant (*p* < 0.05, n = 3). In contrast to the case of NBRC3832, the growth rate of NH04 was not affected by introduction of pGK*ahp*C (Figure 3b). The doubling times of NH04 harboring pGK*ahp*C and pGK::*nuc*MCS were both 9.8 ± 0.3 h at 10°C. At 25°C, introduction of pGK*ahp*C did not significantly affect the growth rate of NBRC3832 and NH04 (data not shown). The doubling times of NBRC3832 harboring pGK*ahp*C and pGK::*nuc*MCS were 3.0 ± 0.2 h and 3.2 ± 0.1 h, respectively, at 25°C, which were not significantly different from each other (*p* < 0.05, n = 3). The doubling times of NH04 harboring pGK*ahp*C and pGK::*nuc*MCS were both 2.0 ± 0.1 h at 25°C.

## Discussion

We isolated a novel food-spoilage bacterium, NH04, from spoiled meat product at 10°C, which causes food quality defects at low temperatures. Although little is known regarding the effects of various stresses on *Leuconostoc* species, they can survive in unfavorable environments such as oxidative and low temperature conditions (Con and Seamus 1986; Salotra et al. 1995; van de Guchte et al. 2002). Most *Leuconostoc* strains grow even at 10°C, and NH04 grows faster than other related strains at both 10°C and 25°C (Figure 1).

A comprehensive analysis of proteins synthesized in NH04 demonstrated that this strain inducibly produces a homolog of alkyl hydroperoxide reductase, AhpC, which likely has a role in anti-oxidative functions at low temperatures (Figure 2). AhpC homologs are widely distributed among prokaryotes, and AhpC shares approximately 40% amino acid sequence identity with thioredoxin peroxidase from yeast, rat, plant, amoebae, nematodes, rodents, and humans (Chen et al. 1998). AhpC homologs define a large family of anti-oxidants present in organisms from all kingdoms, and they protect cells from reactive oxygen species (ROS). ROS such as O_2_^−^, •OH, and H_2_O_2_ cause oxidative damage to cells (Cabiscol et al. 2000). The O_2_^−^ generated during cell growth is typically converted to hydrogen peroxide spontaneously or by the activity of superoxide dismutase (SOD), from which the hydroxyl radical is generated by a metal-catalyzed redox reaction (the Fenton reaction). Hydroxyl radicals react immediately with cellular components and generate organic hydroperoxide (ROOH) that causes cell damage. AhpC catalyzes the reduction of organic hydroperoxide and protects cell components, thus acting as an anti-oxidant against hydroxyl radicals. Since cold stress induces production of ROS (Gocheva et al. 2009), psychrotrophic LAB that induce the expression of anti-oxidative protein(s) may grow rapidly at low temperatures. We demonstrated that the production of AhpC from NH04 in the related strain, NBRC3832, promotes growth at low temperatures (Figure 3). This suggests an important role for AhpC in the growth of LAB at low temperatures. Reduction of organic hydroperoxide may be crucial for LAB to grow at low temperatures. NH04 grows faster than NBRC3832 not only at 10°C but also at 25°C. However, the faster growth of NH04 at 25°C is not likely due to AhpC, because the expression level of AhpC in NH04 at 25°C is much lower than at 10°C and a statistically significant growth improvement of NBRC3832 by overexpression of AhpC was not observed at 25°C.

In this study, due to the lack of a gene disruption system for NH04 and related strains, the involvement of AhpC in the cold adaptation of NH04 could not be shown directly by a loss-of-function analysis. Nevertheless, the results obtained by heterologous expression of AhpC in a related strain strongly support the view that this protein is involved in the growth of LAB at low temperatures. Our results provide new insight into the molecular mechanism of cold adaptation of food-spoilage LAB that cause food quality defects during cold storage. In the future studies, we will examine whether AhpC is involved in the cold adaptation of other food-spoilage LAB, and whether other anti-oxidative substances play a role in cold adaptation. Our findings raise a possibility for the control of food-spoilage related LAB growing at low temperatures by developing an inhibitor against their anti-oxidation system. It may also be possible in the future for food manufacturers to detect food products and manufacturing equipment contaminated with food-spoilage LAB by developing a system to detect AhpC as a marker.
